# Editorial: Impact of Human Learning and Ergonomics on Medical Education in Minimally Invasive Surgery

**DOI:** 10.3389/fsurg.2021.744154

**Published:** 2021-08-20

**Authors:** Ka-Chun Siu, Priscila Rodrigues Armijo, Crystal M. Krause, Gezzer Ortega, Fernando A. M. Herbella

**Affiliations:** ^1^Department of Health & Rehabilitation Sciences, University of Nebraska Medical Center, Omaha, NE, United States; ^2^Department of Surgery, University of Nebraska Medical Center, Omaha, NE, United States; ^3^Department of Surgery, Brigham and Women's Hospital, Harvard Medical School, Boston, MA, United States; ^4^Department of Surgery, Federal University of São Paulo, São Paulo, Brazil

**Keywords:** medical education, human learning, minimally invasive surgeries, patient safety, skills learning

Providing adequate and effective training for health care providers is critical in current clinical practice to ensure the safety of patients. However, the primary limiting factor for trainees to gain enough practice to master the necessary clinical skills is time available for practicing these skills (1). Over the past decade, the innovations in training and technology simulation have offered potential solutions for safe and cost-effective training environments for trainees to master fundamental skills, including motor skills (2–4) and problem-solving (5). One of the main challenges of surgical skills learning is providing an optimal training environment in which to develop skills readiness, maintenance, and retention (6, 7). It is essential to study human learning and ergonomics to assess the use and impact of innovative technology on medical education in minimally invasive surgery and other surgical specialties. In this Research Topic, several studies are presented to demonstrate how the better use of innovative technology, minimally invasive surgery, and learning curve analysis can improve medical education and elevate patient safety in clinical practice.

Wang et al. conducted a retrospective study to compare the efficacy and safety of the conventional anti-Nuss operation with those of a modified anti-Nuss operation using a flexible plate in patients with pectus carinatum. Patients who underwent the modified procedure had a shorter operation duration, postoperative hospitalization, and duration of plate removal surgery. No significant differences were found in clinical outcomes, e.g., incidence of complications, between the two anti-Nuss operations. The innovations in technology in this study provide a good example of the need to advance surgical technology for clinical practice while ensuring the patient safety and care.

Complications can arise after minimally invasive surgery. For instance, postoperative pancreatic fistula is problematic after laparoscopic pancreaticoduodenectomy. Sun et al. examined their clinical experience utilizing a modified Blumgart pancreatojejunostomy (an anastomosis procedure) during laparoscopic pancreaticoduodenectomy. They found the modified Blumgart anastomosis simplified the creation of the pancreatojejunostomy during the laparoscopic pancreaticoduodenectomy, and the hospital length of stay was reduced. This study is an example of how innovations in technology could impact clinical practice and provide a means to improve clinical outcomes.

Qin et al. investigated the improvement of surgical outcomes in patients with early-stage cervical cancer. They compared laparoscopic radical hysterotomy with open abdominal radical hysterectomy and found similar survival and improved surgical outcomes between the two procedures. Laparoscopic radical hysterotomy yields shorter operation time, less blood loss and lower postoperative ileus rates. The comparative clinical outcomes between the two procedures demonstrate alternative surgical options using minimally invasive approach for patients with early-stage cervical cancer. This study supports the use of minimally invasive surgery in clinical practice and the need for more training.

Learning a new surgical skill can be challenging, especially when using a minimally invasive approach. Zhu et al. applied cumulative sum analysis to better explore the learning proficiency in minimally invasive esophagectomy. To compare open esophagectomy with minimally invasive esophagectomy with respect to morbidity and mortality, Zhu et al. investigated the learning curve of lymph node dissection around the recurrent laryngeal nerve in McKeown minimally invasive esophagectomy and evaluated the surgical outcomes. Learning proficiency in precise procedure was assessed by cumulative sum analysis. This study provided educational guidance on how to better train surgeons to perform minimally invasive esophagectomy, showing that the McKeown minimally invasive esophagectomy could be performed proficiently and safety after ~151 cases. Studying human learning in surgery through learning curve analysis provides meaningful evidence to promote medical education and advance minimally invasive surgical skills.

This research special topic highlights how minimally invasive surgery and learning curve analysis can improve medical education through the increased use of innovative technology. However, more rising healthcare providers and surgeons with competent minimally invasive surgical skills are needed to further elevate patient safety in clinical practice, so further research into this area is needed. We believe this Research Topic can serve as a catalyst to simulate more interest in conducting evidence-based research to advance medical education in minimally invasive surgery. Further progress in advancing medical education for clinical providers will result in better outcomes for patients, including lowered morbidity and improved quality of life for patients.

## Author Contributions

K-CS prepared the editorial. FH, CK, PA, and GO reviewed and revised the editorial. All authors finalized and confirmed the editorial.

## Conflict of Interest

The authors declare that the research was conducted in the absence of any commercial or financial relationships that could be construed as a potential conflict of interest.

## Publisher's Note

All claims expressed in this article are solely those of the authors and do not necessarily represent those of their affiliated organizations, or those of the publisher, the editors and the reviewers. Any product that may be evaluated in this article, or claim that may be made by its manufacturer, is not guaranteed or endorsed by the publisher.
